# Post-LSCS uterocutaneous fistula-utility of magnetic resonance imaging in its diagnosis

**DOI:** 10.4274/tjod.galenos.2019.29560

**Published:** 2019-07-03

**Authors:** Mohd Ilyas, Insha Khan, Tariq Gojwari, Musaib Ahmad Dar, Fahad Shafi, Obaid A Shah

**Affiliations:** 1 Sher-I-Kashmir Institute of Medical Sciences, Department of Radiodiagnosis, Srinagar, India; 2 Sher-I-Kashmir Institute of Medical Sciences, Department of Obstetrics and Gynecology, Srinagar, India

**Keywords:** LSCS, uterocutaneous fistula, MRI

## Abstract

The present report describes one of the rarest complications of cesarean section, uterocutaneous fistula, diagnosed on magnetic resonance imaging (MRI). A 37-year-old female with history of lower segment caesarean section (LSCS) four years previously presented with a chief symptom of discharge from the right end of a Pfannenstiel incision and on further evaluation was found to have uterocutaneous fistula arising from the LSCS scar to the right end of the abdominal incision. Uterocutaneous fistula is a rare delayed complication of LSCS and MRI plays a definitive role in the accurate diagnosis and delineation of the tract. The present case highlights that although rare, uterocutaneous fistulae must be kept in mind in patients presenting with discharge from the abdominal incision site and MRI evaluation should be performed in such cases for appropriate delineation of the tract.

**PRECIS:** To identify the possible risk factors for postpartum urinary retention.

## Introduction

Uterine fistulae usually occur between the uterus and bowel or bladder (uterocolic or uterovesical) with uterocutaneous forming the rarest variety of uterine fistulae. The fistulae occur due to postoperative injuries or infections, use of drains, and incomplete closure of incision^(1)^. Uterocutaneous fistulae, being a rare condition, need appropriate diagnosis for which magnetic resonance imaging (MRI) plays the most important role, thereby helping in the proper delineation of the tract to guide the appropriate management^(2)^. The most common cause of uterocutaneous fistula is incomplete closure of the cesarean section wound. Earlier diagnosis can be made using fluoroscopic or cross-sectional modalities^(3)^. Surgical repair is the treatment of choice with preoperative gonadotropin administration showing better outcomes, but few cases may result in hysterectomy^(3)^. We describe this case of uterocutaneous fistula, which occurred 4 years after a caesarean section and was diagnosed using MRI.

## Case Report

**Figure 1:**
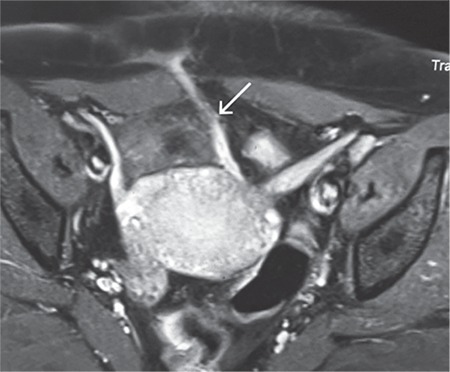
Post-contrast magnetic resonance image showing the enhancing fistulous tract (arrow) from the right end of the abdominal incision traversing through the abdominal cavity to the uterine wall

**Figure 2:**
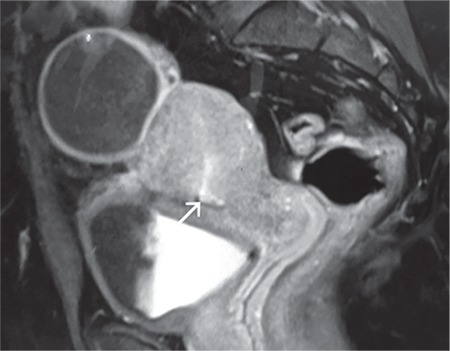
Post-surgical magnetic resonance image after 2 months, showing the LSCS scar (arrow) with no tract demonstrable. Additionally, she developed a simple ovarian cyst, which resolved on its own in 8 weeks LSCS: Lower segment cesarian section

A 37-year old female with a history of lower segment cesarian section (LSCS) performed four years previously presented with the chief symptom of discharge from the incision site. MRI of the pelvis was performed, which revealed an enhancing fistulous tract originating from the site of the lower segment of the cesarean section and traversing through the parietal wall opening at the right edge of abdominal incision scar (Figure 1). The diagnosis of uterocutaneous fistula following LSCS was formulated. After exploratory laparotomy, excision of the tract was performed followed by administration of broad-spectrum antibiotics with postoperative imaging showing no active tract (Figure 2). The patient remains under follow-up and is currently free of symptoms.

## Discussion

During the era of classic cesarean section, a number of cases of uterocutaneous fistula were reported but with the advent of LSCS, the frequency has decreased to a large extent. Most uterocutaneous fistulae owe their origin to infections (e.g. genital tuberculosis) complicating uterine or abdominal scars. Besides LSCS, the causes of uterocutaneous fistulae include post septic abortion, placement of drain, missed uterine perforation following a diagnostic laparoscopy^(4,5)^.

Cases of post-partum hemorrhage wherein B-lynch sutures are placed have high risk of scar dehiscence, which may result in uterocutaneous fistula^(6,7)^. Other rare causes that may result in the formation of uterocutaneous fistula are patients with multiple abdominal myomectomies, history of hysterectomy, and as a primary presentation in underlying gynecologic malignancy such as endometrioid adenocarcinoma, which can predispose the weak cesarean scar to fistula formation^(6,7,8)^. The blood leakage from the incision site during menstruation has been described as pathognomonic of the uterocutaneous fistula^(9)^.

The various modalities that can be used in the diagnosis of uterocutaneous fistula include fistulography with injection of contrast through the skin site, hysterosalpingography with injection of contrast via cervix, computed tomography (CT) scanning, and MRI. Fistulography and hysterosalpingography define the fistulous tract but cannot provide details of its communication with the other intra-abdominal viscera^(1,3)^. CT imaging helps in the proper delineation of the tract after the injection of the contrast agent through the abdominal site, but soft tissue resolution of CT scans is less compared with MRI^(10,11)^. MRI provides good soft tissue resolution, avoids radiation (as all other investigations involve significant radiation doses), and helps in proper delineation of the fistulous tract and its relation to the surrounding viscera. The assessment of other pelvic organs with greater spatial resolution is possible. Intravenous contrast administration shows the enhancement of the granulation tissue along the fistulous tract, which gives a clue about the active status of the fistula. Further, contrast agent can also be administered via the abdominal site as in other investigations to check the patency of the^(12)^.

Hence, MRI forms the best investigative modality in the assessment of uterocutaneous fistulae and other pelvic post-operative complications.
